# Bilateral Traumatic Testicular Dislocation

**DOI:** 10.1155/2018/7162351

**Published:** 2018-05-13

**Authors:** Nubyhélia Maria Negreiro de Carvalho, Ana Clemilda Ximenes Marques, Ivon Teixeira de Souza, Vanessa Guerreiro Soares, Felipe Gomes do Nascimento, Letícia Macedo Pinto, Luccas Victor Rodrigues Dias, Guilherme Carneiro Teixeira

**Affiliations:** ^1^Cirurgia Geral do Hospital Geral de Fortaleza, Fortaleza, CE, Brazil; ^2^Curso de Medicina da Universidade de Fortaleza and Urologista Hospital José Martiniano de Alencar, Fortaleza, CE, Brazil; ^3^Medicina da Universidade de Fortaleza, Fortaleza, CE, Brazil; ^4^Medicina da Unichristus, Fortaleza, CE, Brazil

## Abstract

Traumatic testis dislocation is an uncommon condition and usually occurs after direct impact on the scrotum. We present an uncommon case of bilateral testicular dislocation caused by an automotive accident, which is the most frequently associated cause described in literature today. Although the fact that diagnosis can be made either by physical examination or with the use of basic exams such as Doppler ultrasound, it is not uncommon for cases to go undetected and diagnosed late. In this case, there was a late diagnosis, almost one year after the motorcycle accident. Despite this, studies describe preservation of spermatogenesis even after delayed surgical correction. The treatment can be made by manual reduction, but most of the cases need surgical correction. Therefore, it is always necessary to perform the complete physical examination of the polytrauma patient on the first medical exam, in order to avoid the risk of fertility loss, endocrine dysfunction, and future malignancy.

## 1. Introduction

Testicle dislocation is a quite uncommon event and can be defined as the displacement of one or both normally located testes out of the scrotum. It usually happens after scrotum trauma [1, 2].

Since the first reports of this injury as a result of crushing by wagon wheels, the cause has changed. Motorcycle collisions are the most frequent mechanism cause today [3]. It usually results from straddle injuries sustained in a motorcycle collision, as a result of blunt scrotal injury. A cremaster muscle spasm is a major contributing factor [4–6].

## 2. Case Report

The patient was a 27-year-old male motorcycle driver at the time. He arrived at the emergency on October 31, 2015, after a traffic accident, which resulted in severe polytrauma, nonsurgical cranioencephalic trauma, hemorrhagic contusions, and zygomatic fracture.

The patient was submitted to cranium computerized tomography (CT), which presented hemorrhagic contusions. During the hospital stay, the patient presented convulsive episodes, which were controlled with anticonvulsant drugs. After further evaluation on December 20, 2016, his clinical situation was described as a confused mind status, slowdown gait, and bradypsychia. Another cranium CT was performed which revealed gliosis area, centered midline, ectasia of the ventricles, and zygomatic arch fracture.

There is not any description of physical examination during admission on the hospital discharge report, as well as no description of any procedure as manual reduction of the testicles. The patient's sister reported that he had dislocation of both testicles since the accident has happened, which led to episodes of urinary retention and repetitive urinary tract infections. She informed that to the emergency medical team at the time, but the complaint was not approached. After discharge from hospital stay in the traffic accident event, the patient looked for urology specialized care for an appropriate evaluation.

**Figure 1:**
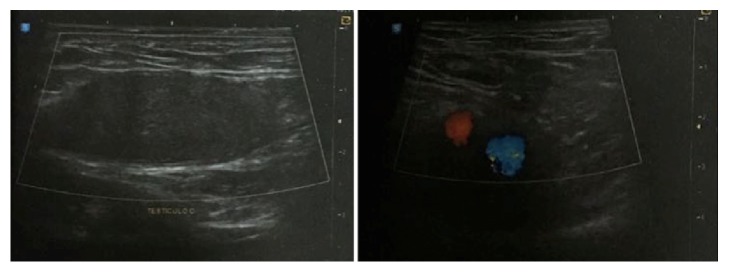
Doppler ultrasound of the right testicle.

**Figure 2:**
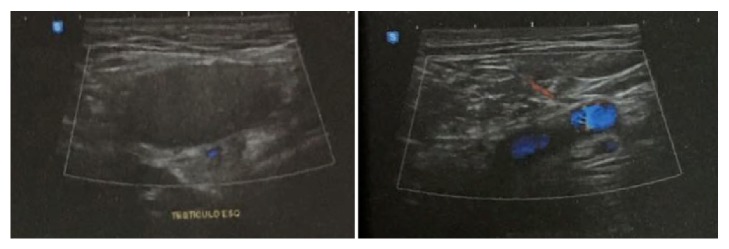
Doppler ultrasound of the left testicle.

In order to have an accurate diagnosis, the patient underwent abdominal and inguinal ultrasound on August 19, 2016, both with normal results, and also underwent a Doppler ultrasound (Figures 1 and 2) of the scrotal sac on the same day, which revealed bilateral cryptorchidism, with no Doppler alterations.

The patient was admitted on March 26, 2017, to surgical correction. At the admission, on physical examination, the patient presented both empty scrotums and the presence of a tender, soft mass on inguinal area, bilaterally.

Manual reduction was not attempted. It was risky because the accident had happened a long time ago.

**Figure 3:**
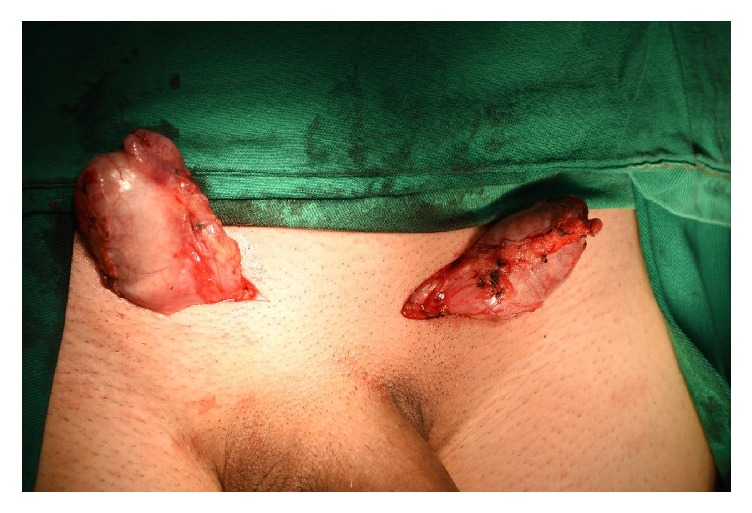
Patient with bilateral testicular dislocation.

**Figure 4:**
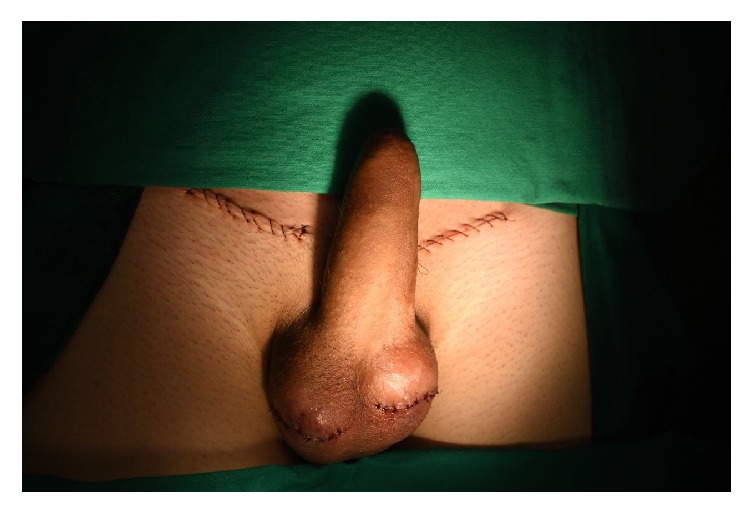
Patient after surgical correction.

In the operation theater, with the patient under spinal anesthesia, an incision of 1,5 inches in each side of the inguinal areal was performed. The groin and the scrotal area were surgically explored, and both testicles were found in the subcutaneous; they were then isolated and each testis presented normal appearance joined to the subcutaneous tissue and with normal length of the spermatic cords. After that, a dissection of a subcutaneous tunnel leading to scrotal sac was performed and a transverse scrotal incision was bilaterally made with 1,1 inches each one. On the right and on the left side, a scrotal subdartos pouch was created and the testicles were, respectively, replaced in the anatomic place in their inside, anatomically, and tension free. Then the skin was sutured with nylon 4-0 and the scrotum with catgut 3-0 (Figures 3 and 4).

The patient was discharged from the hospital on the second postoperative day with ecchymosis on the borders of the scrotal suture.

On the follow-up examination 2 weeks later at the outpatient clinic, the wound had a dry crust in all its extension and the testicles were in anatomical position.

## 3. Discussion

Traumatic testicular dislocation is a displacement of a normally located testis out of the scrotal sac, described for the first time in 1809 by Claubry and it is an uncommon complication of blunt testicular trauma [4–9]. A recent review concluded that less than 200 cases had been notified in indexed literature around the world [10]. It is possible that a lot of cases are underreported.

**Figure 5:**
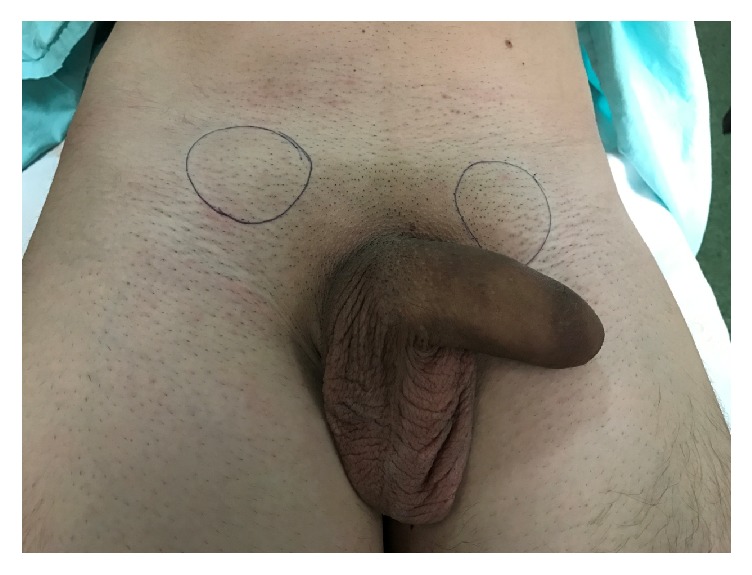
Patient with empty scrotal pouch.

Most cases occur as a result of straddle injuries during high-speed motorcycle accidents when the rider suffers direct traumatic impact on the perineum and scrotum over the fuel tank or handlebar [1, 4]. The shape of fuel tank wedges the groin area, forcibly displacing each testis into superolateral direction [8, 11]. It usually happens just after the injury, but it has been described even 4 days after a scrotal trauma. There is not a specific method to differentiate between truly undescended, retractile testes and those that sustained traumatic dislocation. The clinical history and the physical examination have a crucial part in the investigation. In this special case, there were the antecedents of a motorcycle accident, the hospitalization, and the report of the patient's sister. At the physical examination we observed that the scrotal pouch was normotrophic, which suggests that the patient had a normal development due to the presence of the testes (Figure 5).

Although testicular dislocation is more common unilaterally, it occurs bilaterally in one-third of the cases, approximately [1, 3, 4, 7, 12]. It can also be superficial (into superficial inguinal pouch) or internal (into the external ring in the inguinal canal or in the abdominal cavity) [7, 8]. In 50% of the cases the testicle migrates to the superficial inguinal region. Other reported sites in studies include pubic (18%), penile (8%), canalicular (8%), truly abdominal (6%), perineal (4%), and crural regions (2%) [4, 5, 7]. The ultimate location of testicular dislocation is related to the mechanism of injury, the direction and intensity of the impact, the presence of anatomic abnormalities, and a brisk contraction of the cremaster muscle at the moment of trauma, associated with a secondary cremaster muscle spasm contraction [6, 9, 13, 14].

Testicular dislocation can be the cause of various testicular injuries, which include minor contusions, hematoma, ruptured tunica, and completely shattered testis [5]. Moreover, the presence of this type of injury can be very useful in the course of medicolegal investigation of a fatal motorcycle accident, helping to identify the motorcycle driver and to determine responsibilities in the accident [6].

Spasm of the cremaster muscle is the factor most frequently associated with testicular dislocation, which can retract the testis out of the scrotal sac. Some underlying anomalies as a wide external inguinal ring, an indirect inguinal hernia, and atrophic testis can contribute as predisposing factors [4, 5, 15].

On the physical examination, the empty scrotal sac leads the investigation. Finding a tender, consistent mass with displaced testis in conjunction with an empty hemiscrotum in a patient with no history of orchiectomy or cryptorchidism helps to diagnose this condition [3].

Ultrasonography is usually the first imaging test to be performed and color Doppler ultrasound can help to assess the blood flow of testis to exclude coexisting conditions as testicular rupture, torsion, or epididymal avulsion, and it is also useful for evaluating the vitality of the testicle [4, 15, 16]. In some cases, when the diagnosis is unclear or the testis cannot be recognized neither by physical examination nor by ultrasound, a CT scan may be necessary [4].

Late diagnosis can be observed days or weeks after the accident, but there are reports of more than 10 years of delay [14, 17]. It can happen during polytrauma injuries and in some cases it is demonstrated only in an abdominal CT scan or ultrasonography [7, 8, 11]. Delay in diagnosis can lead to spermatogenic function loss and increased risk of orchiectomy [5, 18].

Differential diagnosis includes undescended testis, retractile testis, or traumatic testis torsion with high-lying testis [7, 8].

A lot of authors state that manual reduction should always be the first treatment for a normal testicle with no coexistent injuries, but only 15% of the cases have a successful result. Factors that are associated with unsuccessful closed reduction include small size of the injury in the spermatic cord layers, the presence of edema, and the possibility of other associated injuries [3, 4, 19], for example, associated testicular torsion or rupture contraindicates closed reduction. In these cases an ultrasound or another image test is necessary before attempting closed reduction [8]. Also, delayed reduction in postpubertal men has been reported to impair spermatogenesis, which is usually detected after 4 months of dislocation [4, 20]. The main risk of delayed reduction is that torsion of the dislocated testis can be missed and an originally viable testis could become gangrenous [21].

Surgical exploration and orchidopexy should be performed early to evacuate the hematoma, repair lacerated tissue, and fix the testicle after repositioning it. Tai et al. postulate that surgical exploration of inguinal and scrotal regions has advantages over manual reduction because it can identify the dislocated testicle and treat coexistent injuries [4]. It also avoids any chance of iatrogenic torsion of testis while doing closed reduction, which needs postreduction Doppler to exclude it [8]. Some studies recommend surgical intervention because coexistent testicular injuries can be present and manual reduction is a poorly successful procedure [4, 22, 23]. Recovery of spermatogenesis has been reported in cases of bilateral dislocation undergoing orchidopexy a lot of years after the injury, which further supports surgery in cases of late diagnosis [1, 4, 20]. Also, the surgery has fast recovery, immediate pain relief, and minimal morbidity [4].

The technique which is used in cases of testis dislocation is the testis fixation at the dartos layer [5]. The main reason for subdartos pouch choice is that fixation with sutures, which traverse the blood-testis barrier, can generate an autoimmune response that leads to eventual infertility. Suture fixation has additional complications. Nonabsorbable sutures are associated with the formation of microabscesses and granulomas, predisposing to chronic testicular pain, while absorbable sutures result in only fine adhesions at the site of placement and thus increase the risk of recurrent torsion [24].

In cases where diagnosis or treatment is delayed, potential complications include torsion, testicular ischemia, diffuse atrophy of seminiferous tubules, severe impairment of spermatogenesis, and acute and chronic discomfort [20, 23, 25]. Infertility may occur due to high temperature exposure leading to reduced spermatids, spermatogonia, and relatively increased Sertoli cells [8, 20]. Early diagnosis and treatment are important to preserve testicular function and to avoid the risk of malignant transformation [3].

## 4. Conclusion

Dislocation of testis is an uncommon injury that, although not acutely life-threatening, carries with it the risk of future malignancy, fertility loss, and endocrine dysfunction [3].

We present a case of bilateral dislocation, which is even more unusual. In this case, the diagnosis took almost one year. Despite the delay in performing the appropriate treatment, both testicles were viable and the surgery had no complications. The patient evolved with clinical improvement.

This case emphasizes the importance of routine bilateral palpation to avoid delay or even undiagnosed testicular dislocation, especially in motorcycle accidents.
